# Effect of PDCA cycle on critical value management in primary healthcare institutions: an interrupted time series study

**DOI:** 10.3389/frhs.2026.1745945

**Published:** 2026-04-02

**Authors:** Xiaolong Cui, Chen Liang, Shijie Jia, Ying Bi, Qing Li, Xiaomin Zhao, Min Wang, Zhongquan Tang, Ting Ou, Xinyu Dai, Jingqing Yao, Yuntao Li, Hong Ding

**Affiliations:** 1 Department of General Medicine, The Second Affiliated Hospital of Nanjing Medical University, Nanjing, Jiangsu Province, China; 2 Emergency Department, The Second Affiliated Hospital of Nanjing Medical University, Nanjing, Jiangsu Province, China

**Keywords:** critical values, healthcare quality improvement, interrupted time series analysis, patient safety, PDCA cycle, primary healthcare institutions

## Abstract

**Background:**

Critical value management represents a core component of medical safety, yet primary healthcare institutions continue to face challenges including non-standardized processes, incomplete documentation, and insufficient IT support. Although the PDCA cycle is widely adopted as a quality management tool, most evaluations of its effectiveness rely on simple pre-post comparisons, failing to distinguish true intervention effects from underlying secular trends.

**Methods:**

We conducted a prospective, multicenter study implementing a 24-month PDCA cycle intervention across 62 primary healthcare institutions in Jiangsu Province. Quality improvement initiatives included implementing a unified Critical Value Reporting Protocol, standardizing logbooks, and establishing monitoring mechanisms. We employed an interrupted time series model to analyze 24 months of data, assessing both immediate and sustained intervention effects.

**Results:**

Following PDCA implementation, the standardized critical value management rate increased from a pre-intervention average of 93.8% to 98.9%. Interrupted time series analysis revealed a significant immediate improvement (OR = 1.721, *p* = 0.012) and a progressively strengthening trend effect (OR = 1.298, *p* < 0.001). The model demonstrated excellent fit with no residual autocorrelation, and statistical inferences based on robust standard errors proved reliable.

**Conclusion:**

The PDCA cycle effectively enhances the standardized management of critical values in primary healthcare settings. Interrupted time series analysis provides a scientific foundation for evaluating its effectiveness. This model offers substantial operational practicality and scalability, contributing to continuous improvement in healthcare quality.

## 1 Introduction

Critical values refer to abnormal findings in diagnostic tests that indicate a patient may be in a life-threatening condition. These results require immediate notification to the attending physician, who must then promptly initiate appropriate interventions to ensure patient safety (1, 2). Consequently, critical value management constitutes a vital component of healthcare administration and is explicitly mandated by the *Key Points of Core Medical Quality and Safety Systems* (3). Peer-reviewed studies have also emphasized the importance of standardized critical value reporting and closed-loop management (4, 5).

However, in clinical practice, particularly within primary care institutions operating with limited management resources, the standardization of critical value management continues to face significant challenges (6). Widespread issues such as inconsistent adherence to protocols, non-standardized documentation, and the absence of an integrated digital closed-loop system frequently result in reporting delays, information loss, and even failure to initiate appropriate interventions. These deficiencies consequently pose substantial risks to patient safety (7). To address these challenges, the PDCA cycle has been applied in hospital-based studies to improve critical value management processes (8–10). Through its systematic approach of Plan, Do, Check, and Act, it enables healthcare institutions to identify issues, implement improvements, and sustain successful outcomes (11). However, despite its theoretical appeal, many existing studies on the PDCA cycle suffer from methodological limitations in effectiveness evaluation (12). These studies often rely on simple pre- vs. post-intervention comparisons, merely comparing indicator values at isolated time points before and after implementation. This approach cannot distinguish whether the observed improvements represent a true intervention effect or merely reflect underlying secular trends, thereby failing to provide robust causal inference regarding the actual effectiveness of the PDCA cycle (13). To address these methodological limitations, this study introduces a more rigorous evaluation approach: Interrupted Time Series Analysis (ITSA). This method is particularly suitable for assessing quality improvement initiatives implemented at a specific point in time. By collecting data at multiple time points before and after the intervention over an extended period, ITSA enables the analysis of the intervention's actual impact on outcomes while accounting for underlying trends (14–16).

This study integrates Interrupted Time Series Analysis (ITSA) with PDCA cycle methodology to evaluate the standardized critical value management rate across multiple primary healthcare institutions. By implementing standardized PDCA quality improvement tools across 62 institutions in Jiangsu Province and employing ITSA for scientific evaluation, we aim to rigorously assess the effectiveness of PDCA in enhancing critical value management compliance. Our objectives extend beyond validating this improvement model's efficacy to establishing a comprehensive operational framework for medical quality control in Jiangsu's primary care settings, thereby providing a replicable model for other regions.

## 2 Materials and methods

### 2.1 Study subjects

This study employed a prospective, multicenter design conducted over a 24-month period from October 2023 to September 2025. A multi-stage, non-random quota sampling method was utilized. Within each participating prefecture-level city, we implemented pre-established sample institution quotas. Considering Nanjing's status as the provincial capital, along with its institutional diversity and leading role in regional healthcare, we allocated it a larger sample size of 13 institutions. Wuxi City received 5 allocations due to its robust pre-existing digital infrastructure. The remaining 11 prefecture-level cities were each assigned 4 institutions to ensure adequate geographical coverage, resulting in a final total of 62 institutions. Details are presented in Table 1. Inclusion Criteria: 1) Outpatient department (inpatient department optional); 2) Complete Hospital Information System (HIS) and Laboratory Information System (LIS); Picture Archiving and Communication System (PACS) required for institutions with imaging departments; 3) Public healthcare institutions; 4) Voluntary participation. Exclusion Criteria: 1) Non-public healthcare institutions; 2) Lack of required information systems; 3) Unwilling to comply with data reporting protocols.

**Table 1:** Basic characteristics of the 62 included primary healthcare institutions.

Category	Item	Number (n)	Percentage (%)
Region	Nanjing City	13	21.0
	Wuxi City	5	8.1
	Other 11 Prefecture-level Cities	44	70.9
Hospital Grade	Level 1	52	83.9
	Level 2	10	16.1
Institution Type	Community Health Center	35	56.5
	Township Health Center	27	43.5
Bed Capacity	0–50 Beds	16	25.8
	51–100 Beds	23	37.1
	>100 Beds	23	37.1
Annual Outpatient Visits	<100,000	14	22.6
	100,000–200,000	24	38.7
	>200,000	24	38.7

Summary of institutional characteristics from the 62 participating primary healthcare institutions in Jiangsu Province, China. Data are presented as frequency counts and corresponding percentages.

### 2.2 Data collection

In accordance with the requirements for standardized critical value management stipulated in the nationally issued *Key Points of Core Medical Quality and Safety Systems*, critical values must be managed through a standardized closed-loop process. This includes identification and reporting, notification, receipt, handling, logbook documentation, and feedback, with full traceability of all relevant records required throughout the entire process (3). Based on the *Key Points of Core Medical Quality and Safety Systems*, we developed a unified *Critical Value Management Protocol*. At each selected institution, one data collector was trained to thoroughly study and master this protocol. These collectors are required to extract data from their respective healthcare information systems and critical value logbooks, then submit it monthly to the Quality Control Center's data reporting platform over a 24-month period. To ensure data accuracy, this study implemented a comprehensive quality control protocol including: standardized training for all data collectors to ensure uniform data standards; designated quality control specialists conducting monthly verification and traceability checks for abnormal values; regular unannounced audits with cross-verification among information systems, critical value logbooks, and patient medical records before final dataset compilation.

### 2.3 Implementation of the PDCA cycle

**Figure 1:**
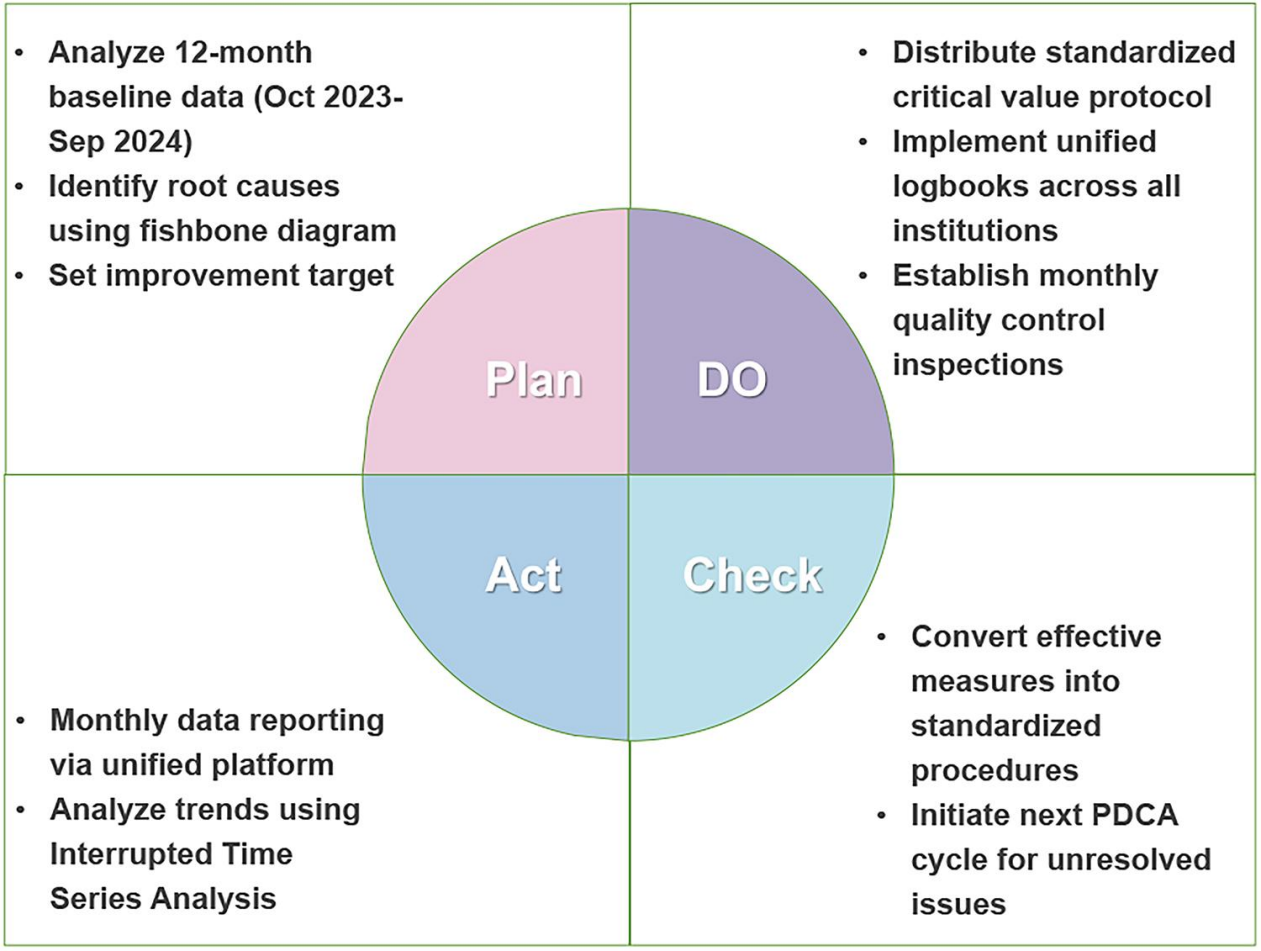
PDCA cycle for critical value management improvement. Flowchart of the PDCA (Plan-Do-Check-Act) cycle implemented to standardize critical value management processes in primary healthcare institutions.

The study will initiate the PDCA cycle in late September to early October 2024 to implement quality improvement initiatives targeting the standardized critical value management rate. The specific PDCA process is shown in Figure 1. Specifically, the intervention package included unified management protocols, standardized logbook use, staff training and supervision, monthly online audits, unannounced on-site inspections, and quarterly feedback for iterative refinement.

#### 2.3.1 P (plan) phase

A dedicated quality control team was established, comprising healthcare administrators, clinicians and nurses from clinical departments, the Director of the Laboratory Medicine Department, the Director of the Imaging Department, and other relevant personnel. These members typically hold concurrent positions in hospital quality management roles and possess specialized knowledge and skills in healthcare quality management. In September 2024, the quality control team conducted a comprehensive survey of all 62 institutions to establish baseline critical value reporting conditions and identify systemic issues in critical value management. The team identified common problems and completed the baseline data collection for standardized critical value reporting rates by the end of September 2024. The dataset encompasses the total number of critical values and standardized management cases across all 62 institutions during the 12-month period from October 1, 2023, to September 30, 2024. The study data revealed that the overall standardized management rate of critical values across the 62 healthcare institutions during the 12-month baseline period was only 93.8%. According to the national “Primary-level Healthcare Quality Improvement Campaign,” the target rate for standardized critical value management in primary healthcare institutions is 100%, indicating that the baseline results fell short of the required standard (17). The baseline investigation identified the top three issues in non-compliant critical value management as follows: 1) Improper critical value log design; 2) Missing, retroactive, or incomplete documentation; 3) Absence of standardized critical value criteria/protocols. The detailed ranking of common issues is presented in Table 2.

**Table 2:** Common issues with critical value documentation.

Rank	Common issue	Number of institutions	Specific manifestations (and number of institutions)
1	Improper Critical Value Log Design	17	Logs lacked critical fields (e.g., “Intervention,” “Intervention Time,” “Intervention Result,” “Receipt Time,” “Attending Physician”). (10 institutions)
			Logs were not bound, allowing pages to be easily added or removed. (7 institutions)
2	Missing, Retroactive, or Incomplete Entries	17	Evidence of retroactive entries or additions after missed reports. (3 institutions)
			Excessively brief or chronologically incomplete records. (4 institutions)
			Clinical departments failed to log critical values from diagnostic departments (e.g., Imaging, ECG). (3 institutions)
			Entries had unsigned alterations or contained blank pages. (2 institutions)
			Incomplete critical value records (e.g., only contained lab data, missing other departments). (4 institutions)
			Discrepancies between logbook data and the official reporting platform data. (1 institution)
3	Absence of Standardized Criteria/Protocols	12	No hospital-wide unified critical value standards, or standards not affixed to the logbook's front page. (3 institutions)
			Outpatient logs were not separated from those for diagnostic departments. (4 institutions)
			Mismatched critical value items between clinical and diagnostic departments. (2 institutions)
			Disorganized logging or excessive number of logbooks (e.g., one per staff), hindering management. (2 institutions)
			The critical value reporting process is chaotic/disorganized. (1 institution)
4	Poor Logbook Management	8	Logs were not bound, allowing pages to be easily added or removed. (4 institutions)
			No critical value logbook for clinical departments whatsoever. (1 institution)
			Logbooks were dilapidated. (3 institutions)
5	Logbook Format Issues	6	Log format was not aligned with actual workflow or was outdated. (4 institutions)
			Log content directly copied lab department items, not adapted for clinical use. (2 institutions)

Categorization and frequency analysis of documentation deficiencies identified during the baseline audit. Specific manifestations are listed with institutional counts in parentheses.

**Figure 2:**
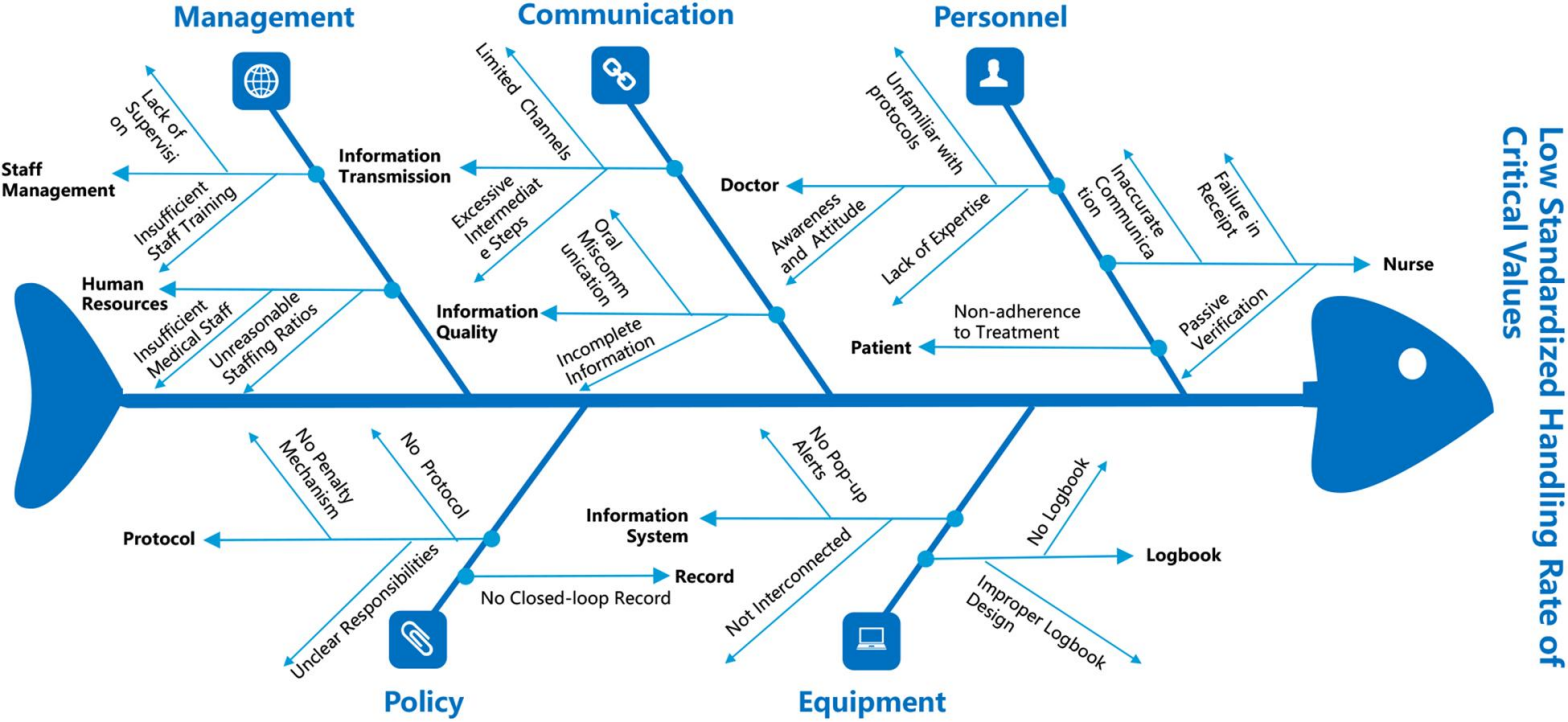
Fishbone diagram of root cause analysis for critical value management quality issues in primary healthcare institutions. This fishbone diagram (cause-and-effect diagram) systematically analyzes the root causes affecting the quality of critical value management in primary healthcare institutions. The analysis categorizes contributing factors into five main dimensions.

The research team organized a special seminar focused on using the PDCA methodology to enhance the standardized critical value management rate in primary healthcare institutions. During the seminar, participants reviewed the baseline findings and conducted a brainstorming session using a fishbone diagram to systematically identify all potential root causes contributing to “non-compliant critical value management”. Through the thematic discussions, a consensus was reached, identifying three critical contributing factors: inconsistent critical value management protocols, non-standardized critical value logbooks, and inadequate supervision of critical value practices. Based on this analysis, a targeted improvement plan was formulated, aimed at increasing the standardized critical value management rate to over 98%. The fishbone diagram illustrating these root causes is presented in Figure 2.

#### 2.3.2 D (do) phase

This study implemented targeted interventions addressing the three key issues identified through root cause analysis. First, to tackle the “inconsistent critical value management protocols,” the developed *Critical Value Reporting Protocol* was distributed to all 62 institutions, specifying standardized procedures for the entire workflow and defining the scope of reportable critical values. To address the issue of “non-standardized critical value logbooks,” standardized logbook samples were demonstrated to all 62 institutions. Each institution was required to distribute these unified logbooks, containing essential components such as cover pages, documented items, timestamps, reporting personnel, receiving personnel, and actions taken. Finally, to address the challenge of “inadequate supervision,” quality control specialists were assigned to perform monthly reviews of reported data. Additionally, the quality control team regularly conducted unannounced on-site inspections, with results compiled into corresponding reports for internal circulation. This process established an accountability framework to ensure the effective implementation of all targeted measures.

#### 2.3.3 C (check) phase

This study established a dual verification system combining online and offline methods, comprising monthly online reviews and regular unannounced on-site inspections. The online component involved collecting core indicators of standardized critical value management rates from all institutions through a unified platform, with continuous monitoring of abnormal values. Institutions exhibiting unusually high or low values were required to provide explanatory documentation and supporting evidence upon follow-up inquiries. Offline, the Quality Control Center organized expert teams to conduct biannual unannounced on-site inspections. These verifications involved cross-checking system data against medical records to ensure data authenticity and conducting individual interviews with randomly selected healthcare staff to assess the implementation of the standardized critical value management process. Finally, quarterly quality control reports were generated from the online data, with each inspection producing a supervisory report. All findings were disseminated to the 62 institutions, providing continuous feedback to all participating facilities.

#### 2.3.4 A (act) phase

Based on the findings from the verification phase, this study developed a systematic strategy to sustain the achieved improvements and facilitate their adoption across a broader range of institutions. Specifically, the following three approaches were implemented: First, the tools and processes proven effective in practice for improving the standardization rate were systematically compiled into a management framework for implementation throughout the province. This ensures that the quality improvement measures are sustained and integrated into the daily operations of each institution. Second, we systematically summarized the PDCA-based model for improving the standardized critical value management rate developed in this study, and are promoting its application to other medical quality improvement initiatives. Simultaneously, we recommend that all primary healthcare institutions adopt the PDCA cycle for internal medical quality enhancement. Third, unresolved issues identified during the verification phase, such as information systems not yet enabling closed-loop operations, have been designated as priority topics for the next PDCA cycle. This approach establishes an ongoing improvement mechanism integrating PDCA and ITSA methodologies, thus creating a sustainable cycle of evaluation, refinement, and re-evaluation to drive continuous advancement in medical quality.

### 2.4 Statistical analysis

The study employed an interrupted time series analysis to evaluate the impact of the PDCA cycle intervention on the standardized critical value management rate in primary healthcare institutions (18). The study variable was the monthly “standardized critical value management rate,” defined as the proportion of critically managed values to the total number of critical values during the same period. Given that the variable represents proportional data with significant over-dispersion (dispersion parameter = 2.445), we constructed a segmented regression generalized linear model based on a quasi-binomial distribution. The core independent variables in the model included: time (a continuous variable measured in months, used to assess the pre-intervention baseline trend), intervention phase (a binary variable coded as 0 for pre-intervention and 1 for post-intervention, evaluating the immediate level change following the intervention), and post-intervention time trend (assessing the change in trends before and after the intervention). To account for fluctuations in sample size, the model incorporated an offset term with the log denominator. During the model diagnostics phase, we assessed autocorrelation by visually examining the autocorrelation function plot of residuals and statistically validating it using the Ljung-Box test. The results indicated no significant autocorrelation structure in the residuals across all lag orders, with the ACF plot showing no spikes exceeding the confidence interval and the Ljung-Box test yielding a *p*-value of 0.401 (19). Considering the inherent heteroscedasticity of proportional data and the over-dispersion present in the model, we employed heteroscedasticity-robust standard errors for all statistical inferences. This approach ensures the standard errors of parameter estimates remain robust against potential variance fluctuations, with final statistical significance determined based on these robust standard errors.

All statistical analyses were performed using R (version 4.3.1) with the significance level set at *α* = 0.05. The regression coefficients from the model will be converted to odds ratios with corresponding 95% confidence intervals.

## 3 Results

### 3.1 Key statistical findings and model specification

The study included 24 months of continuous data from 62 primary healthcare institutions in Jiangsu Province between October 2023 and September 2025, comprising a total of 28,340 critical value records. The overall average standardized management rate during the 12-month pre-intervention period was 93.8%, which increased to 98.9% after implementing the PDCA cycle, achieving the predetermined target. A quasi-binomial interrupted time series model was employed to more rigorously evaluate the impact of the PDCA cycle intervention on the standardized critical value management rate in primary healthcare institutions. The model was constructed using monthly data from 24 consecutive time points, with the specification as follows:

$$\mathrm{Logit}\;(P)\;=\;\beta_{0}\;+\;\beta_{1}\;\times \;\mathrm{time}\;+\;\beta_{2}\;\times \;\mathrm{intervention}\;+\;\beta_{3}\;\times \;\mathrm{post}\_\mathrm{trend}\;+\;\mathrm{offset}(\log\_\mathrm{denominator})$$

**Figure 3:**
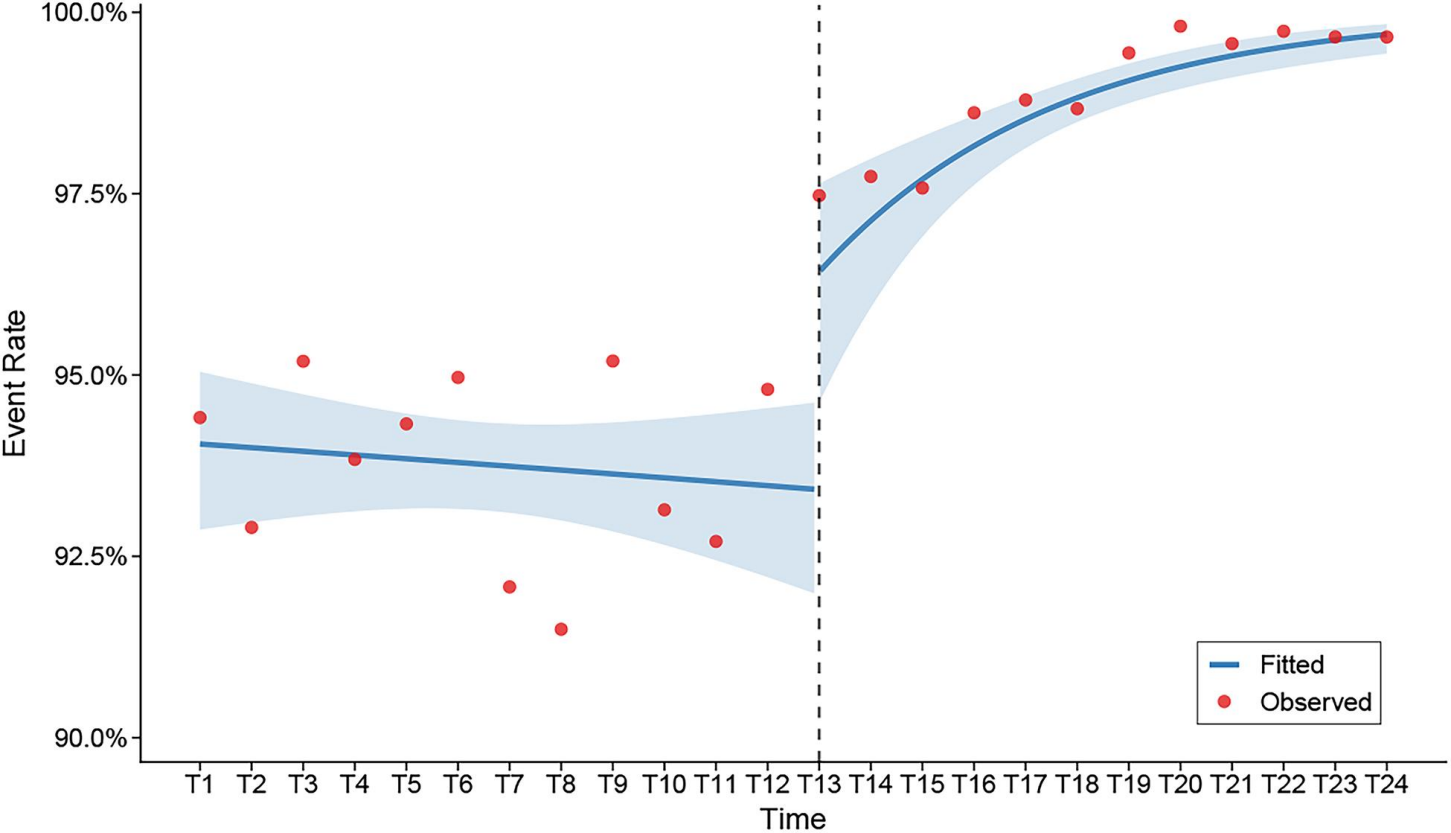
Interrupted time series analysis of critical value management rates before and after PDCA cycle intervention. Red dots represent the actual observed monthly critical value management rates; blue solid line indicates the fitted values from the quasibinomial interrupted time series model; blue shaded area represents the 95% confidence interval; vertical dashed line indicates the start time of PDCA cycle intervention (T13).

Detailed definitions of each model component are provided in Table 3. The interrupted time series analysis using heteroscedasticity-robust standard errors demonstrated statistically significant effects of the PDCA cycle intervention (Table 4). The immediate intervention effect coefficient was 0.543 (OR = 1.721, 95% CI: 1.121–2.641, *p* = 0.012), indicating a significant immediate improvement in the standardized critical value management rate following implementation. The trend effect coefficient was 0.261 (OR = 1.298, 95% CI: 1.131–1.490, *p* < 0.001), demonstrating progressively strengthening intervention effects over time. The non-significant baseline time trend (coefficient = −0.012, OR = 0.988, 95% CI: 0.917–1.064, *p* = 0.503) confirmed the absence of significant natural variation in the critical value management rate before the intervention. As shown in Figure 3, the interrupted time series plot visually demonstrates the trend changes in standardized critical value management rates before and after the intervention. The pre-intervention rates remained relatively stable across time points, while a marked upward trend emerged following implementation, confirming both the effectiveness of the PDCA cycle intervention and the accuracy of model predictions. The immediate post-intervention change showed an increase from 94.8% to 97.5%, representing an absolute improvement of 2.7 percentage points and a relative increase of 2.8%. The statistical model results indicate that this multicenter PDCA cycle quality improvement model effectively enhances the overall standardized management of critical values in primary healthcare institutions, demonstrating not only immediate improvements but also sustained long-term enhancement.

**Table 3:** Definition and statistical properties of interrupted time series model components.

Component	Symbol	Definition
Dependent variable	P	Critical value compliance
Link function	Logit	Logit transformation function
Offset term	log_denominator	Log of denominator as offset
Intercept	β_0_	Baseline level parameter
Time trend	*β*_1_	Pre-intervention trend coefficient
Intervention effect	*β*_2_	Immediate intervention effect coefficient
Trend effect	*β*_3_	Post-intervention trend change coefficient
Time variable	time	Continuous time series, 1–24
Intervention variable	intervention	Intervention time indicator, 0 = pre, 1 = post
Trend variable	Post-trend	Post-intervention time trend
Model distribution		Quasibinomial distribution

Mathematical specification and operational definitions of parameters in the quasi-binomial interrupted time series model. The model structure follows: Logit(P) = *β*_0_ + *β*_1_ × time + *β*_2_ × intervention + *β*_3_ × post-trend + offset(log_denominator).

**Table 4:** Results of the interrupted time series regression analysis.

Parameter	Coef (*β*)	z-value	OR	95% CI for OR	*p*-value
Intercept (*β*_0_)	−4.280	−34.339	0.014	(0.011, 0.018)	<0.001
Time trend (*β*_1_)	−0.012	−0.670	0.988	(0.952, 1.024)	0.503
Intervention (*β*_2_)	0.543	2.523	1.721	(1.129, 2.625)	0.012
Trend effect (*β*_3_)	0.261	7.176	1.298	(1.209, 1.395)	<0.001

Parameter estimates from the interrupted time series regression analyzing PDCA cycle intervention effects. OR, odds ratio; CI, confidence interval. Statistical significance assessed at *α* = 0.05.

### 3.2 Key statistical findings and model specification

**Figure 4:**
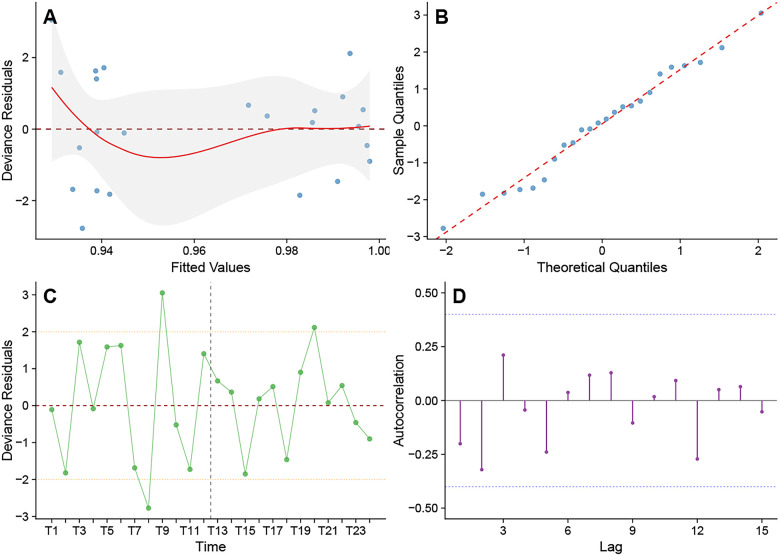
Diagnostic plots for the quasibinomial interrupted time series model. **(A)** Residuals vs fitted plot showing the distribution of deviance residuals against fitted values; **(B)** Normal Q-Q plot for testing normality of residuals; **(C)** Residuals vs time plot showing the temporal pattern of residuals; **(D)** Autocorrelation function (ACF) plot for testing residual autocorrelation.

The model fit results and diagnostic checks for the interrupted time series analysis demonstrate satisfactory statistical properties and reliability. The model explains 92.8% of the deviance (pseudo-R^2^ = 0.928), indicating strong explanatory power. To address significant over-dispersion (dispersion parameter = 2.445), the analysis utilized a quasi-binomial distribution with heteroscedasticity-robust standard errors, ensuring valid statistical inference. Visual inspection of the Deviance residuals using the Autocorrelation Function (ACF) plot, combined with the Ljung-Box test, revealed no significant autocorrelation across all lag orders (Ljung-Box test *p* > 0.05). The ACF plot displayed no significant spikes extending beyond the confidence boundaries (Figure 4), indicating absence of substantial residual autocorrelation and thus meeting the model's independence assumption. Model comparison analysis revealed that when log_denominator was included as a standard covariate, its coefficient was not statistically significant (Wald test based on robust standard errors: *p* = 0.812). Nevertheless, based on the theoretical framework for proportional data and methodological considerations regarding denominator control, we ultimately incorporated it as an offset term in the model to ensure appropriate modeling of the standardized critical value management rate. The model diagnostic plots, including the residual vs. fitted values plot, Q-Q plot, residual time series plot, and ACF plot, collectively indicated satisfactory fulfillment of the model assumptions, further validating the model's applicability (Figure 4). Based on comprehensive diagnostic evaluations, the selected interrupted time series model demonstrated adequate fit to the data, and the statistical inference results are considered valid and reliable.

## 4 Discussion

### 4.1 Advantages of interrupted time series analysis in healthcare quality assessment

This study utilized an interrupted time series analysis model to evaluate the effectiveness of the PDCA cycle in improving the standardized critical value management rate. This methodological approach offers distinct advantages over traditional quality assessment methods (20–22). Traditional pre-post studies typically compare differences at single time points before and after an intervention, making it impossible to distinguish whether observed improvements represent true intervention effects or merely reflect underlying secular trends (23, 24). In contrast, interrupted time series analysis enables precise identification of both immediate level changes and sustained trend modifications following the intervention by utilizing data from 62 institutions over 24 months, thereby providing stronger evidence for causal inference (25–27). Our findings demonstrate a non-significant pre-intervention baseline trend (*p* = 0.503), effectively ruling out natural improvement in the critical value management rate as a confounding factor. The model detected both a significant immediate level change (OR = 1.721, *p* = 0.012) and sustained trend enhancement (OR = 1.298, *p* < 0.001), indicating that the PDCA cycle not only generated immediate effects but also produced progressively strengthening impacts over time. The interrupted time series methodology provides valuable reference for scientific evaluation of primary healthcare quality improvement, facilitating the transition from experience-based approaches to data-driven decision-making in healthcare quality management (28, 29).

### 4.2 Mechanism of PDCA cycle in improving standardized critical value management rate

This study confirms that implementing the PDCA cycle effectively enhances the standardized critical value management rate in primary healthcare institutions. Its success primarily stems from providing a systematic, goal-oriented, and structured workflow (30–32). During the planning phase, current state analysis and root cause identification pinpointed three critical issues: lack of standardized protocols, non-compliant documentation practices, and insufficient oversight mechanisms. This diagnostic work provided clear direction for subsequent interventions, ensuring targeted improvement strategies. In the implementation phase, the introduction of a unified Critical Value Reporting Protocol, standardized logbooks, and systematic monitoring procedures collectively established a comprehensive and standardized management framework. These coordinated measures effectively addressed the inconsistency in critical value management standards and established executable workflows, ensuring successful implementation across all 62 institutions. During the check and act phases, the integrated online-offline verification and feedback mechanism enabled timely problem identification and resolution, while systematically transforming effective solutions into standardized protocols. This created a sustainable quality improvement cycle. The demonstrated model provides primary healthcare institutions with a replicable framework for achieving continuous quality enhancement (33).

### 4.3 The urgent need for digital critical value management in primary healthcare institutions

While this study has demonstrated significant improvement in critical value compliance rates through PDCA cycle implementation, our implementation process revealed substantial deficiencies in the digital infrastructure supporting critical value management at primary healthcare institutions. Most institutions still rely on traditional management modes dependent on manual logging and communication requiring direct human involvement. This approach not only increases the workload of healthcare staff but also inevitably compromises the timeliness and accuracy of critical value reporting. In today's rapidly evolving digital healthcare landscape, such outdated management practices require urgent transformation, and establishing information technology based critical value management systems should be prioritized (34). A robust digital system should incorporate the following functionalities: automated identification and transmission of critical values, continuous monitoring of processing status, automatic documentation of all procedural steps, and timely alerts for unresolved cases exceeding time limits. Such a system would not only enhance operational efficiency but also ensure complete process management of critical values (35, 36). Furthermore, digital systems enable more efficient quality supervision, allowing administrators to promptly monitor the critical value management status across institutions and timely identify and resolve issues. We recommend that healthcare quality management departments increase investment and accelerate the development of information technology infrastructure in primary healthcare institutions, thereby providing technological support for enhanced medical quality.

This study has several limitations. First, the interrupted time series analysis used aggregated monthly data from 62 institutions rather than institution-level panel data, which could not control for heterogeneity between institutions and only evaluated the overall level across all 62 institutions. Future research should employ multilevel models or institution-level panel data analytical approaches to provide more robust evidence. Furthermore, as the critical value management rate approaches its theoretical maximum, accurate prediction of long-term trends becomes challenging. Subsequent efforts should establish 100% compliance as an essential management objective to drive continuous quality improvement. Future research should focus on institutions consistently failing to achieve complete compliance, employing qualitative methods such as in-depth interviews to identify underlying barriers and develop more targeted intervention strategies.

## 5 Conclusion

This study employed an interrupted time series analysis to evaluate the effectiveness of the PDCA cycle in improving the standardized critical value management rate in primary healthcare institutions. The results demonstrate that this quality management model significantly enhances the standardization level of critical value management (37, 38). Through the complete implementation of the PDCA cycle, standardized critical value management procedures were established and oversight mechanisms were enhanced. This systematic improvement demonstrates both applicability and operational feasibility in primary healthcare settings, offering practical reference value for continuous advancement in healthcare quality management (39). The study confirms the demonstrated utility of this approach in enhancing standardized critical value management and merits broader implementation across other primary care settings.

## Data Availability

The original contributions presented in the study are included in the article/Supplementary Material, further inquiries can be directed to the corresponding authors.
